# Different beta-blockers for preventing arrhythmic events in patients with long QT syndrome: a network meta-analysis

**DOI:** 10.3389/fmed.2025.1730755

**Published:** 2025-12-16

**Authors:** Youxu Jiang, Lihua Zhang, Haiyan Xiong, Yuan Li, Chang Su, Mingna Zhu, Yinchuan Zhu

**Affiliations:** 1 Department of Cardiology, The Second Affiliated Hospital of Zhengzhou University/Center for Medical Experiment, The Second Clinical Medical School of Zhengzhou University, Zhengzhou, Henan, China; 2 Nanchang University Queen Mary School, Nanchang, Jiangxi, China

**Keywords:** Long Q-T syndrome, beta-blockers, LQT1/2/3, cardiac events, dose–response relationship

## Abstract

**Objective:**

This study aims to compare the efficacy of different types of beta-blockers in preventing arrhythmias in patients with long QT syndrome (LQTS), and simultaneously analyze the relationship between dose and effect within each drug by incorporating dose as a continuous covariate.

**Methods:**

A systematic search was conducted in the electronic database, with the cutoff date set at July 20, 2025. The search scope was limited to observational studies that compared at least two drug strategies. A Bayesian arm random-effects network meta-analysis (NMA) was performed, in which the daily equivalent dose (mg/day) was included as a continuous covariate to explore the dose–response relationship.

**Results:**

This analysis included 5,692 patients with LQTS from 10 cohort studies. In LQT1, nadolol was superior to propranolol (RR = 0.59, 95% CI: 0.39–0.90) and had the highest probability of being the optimal treatment (SUCRA = 0.92). Dose–response trends for all drugs were non-significant (all *p* > 0.05). For LQT2, no beta-blocker showed significant superiority, though nadolol (SUCRA = 0.78) and propranolol (SUCRA = 0.65) were ranked highest; nadolol alone demonstrated a significant dose–effect relationship (slope = −0.01763, *p* = 0.010). In LQT3, no active treatment differed significantly from atenolol, yet SUCRA values favored nadolol (0.85) and propranolol (0.75). A significant dose–response was observed for nadolol (slope = −0.02136, *p* = 0.023). An unexpected risk reduction was noted for placebo versus atenolol (RR = 0.63, 95% CI: 0.42–0.93, *p* = 0.02), likely due to baseline imbalances. Evidence consistency (node split *p* > 0.38) and sensitivity analyses supported the robustness of these findings.

**Conclusion:**

Nadolol demonstrates significant and consistent clinical advantages in LQT1 patients and should be considered as first-line therapy for this subtype. In LQT2 patients, nadolol not only shows a superiority trend but also has a significant dose–effect relationship, supporting its clinical priority; in LQT3 patients, nadolol exhibits a significant dose-dependent risk reduction trend, though no statistically significant differences in efficacy were observed among all beta-blockers. Additionally, LQT3 patients should be cautious of the potential risks associated with atenolol.

## Introduction

Long Q-T syndrome (LQTS) is a single-gene hereditary cardiac ion channel disorder characterized by prolonged QT intervals, abnormal T waves, and torsade de pointes (TdP) on electrocardiograms, with recurrent syncope, seizures, and even sudden death as clinical features (1, 2). The prevalence of LQTS is approximately 1 in 2,500, with an average age of onset of 14 years (3). Although the overall prevalence of LQTS is not high, the high incidence of sudden cardiac death imposes a significant disease burden and economic burden on families and society (4).

Approximately 70% of LQTS patients have identifiable genetic defects (5). To date, 17 subtypes have been identified, with these genes encoding cardiac voltage-gated potassium, sodium, and calcium ion channel proteins, as well as related factors and membrane regulatory proteins (6). Among these, individuals carrying mutations in the KCNQ1 (LQT1), KCNH2 (LQT2), and SCN5A (LQT3) genes account for 75% of all cases (7). Different subtypes not only have fundamentally different pathophysiological mechanisms but also exhibit significant heterogeneity in their response to beta-blocker therapy. Beta-blockers are currently the first-line treatment for preventing arrhythmic events in LQTS (8). However, differences in key pharmacologic parameters such as receptor affinity, lipophilicity, and central nervous system penetration among commonly used beta-blockers may lead to significant variations in efficacy across different subtypes (9, 10). Additionally, there are currently no randomized controlled trials evaluating the efficacy of different beta-blockers, with most studies being single-center, small-sample observational studies. Additionally, existing observational studies only compare the overall efficacy of different beta-blockers, lacking dose–response relationship analysis that incorporates dose as a continuous covariate. This makes it impossible to clarify whether the efficacy of the same drug varies with dose and further limits the precision of clinical dose recommendations.

To address this critical evidence gap, this study will for the first time employ a network meta-analysis approach to systematically integrate direct and indirect evidence, quantitatively compare the relative efficacy of commonly used beta-blockers in preventing arrhythmic events across LQT1/LQT2/LQT3 subtypes, establish subtype-specific treatment rankings (SUCRA values), identify the optimal drugs for each subtype, and use node splitting to test the consistency of evidence across different subtypes. The results of this study will provide direct evidence to support precision medication for LQTS, driving a shift in clinical practice from “one-size-fits-all” drug selection to “individualized drug selection.”

## Materials and methods

### Data sources and search strategy

A systematic literature search was conducted using the following databases: PubMed, Web of Science, Embase, and the Cochrane Collaboration Database. The following Medical Subject Headings (Mesh) and standard search terms were used: (“Long QT Syndrome” [Mesh] OR LQTS”[Title/Abstract]) AND (“Adrenergic Beta-Antagonists”[Mesh] OR “Adrenergic Beta-Receptor Blockader”[Title/Abstract]) AND (“Arrhythmias, Cardiac”[Mesh] OR “Arrhythmia”[Title/Abstract]). The search scope covers the creation date of each database up to July 20, 2025. Literature screening is limited to English-language publications. The design, implementation, and reporting of this study strictly adhere to the Preferred Reporting Items for Systematic Reviews and Meta-Analyses of Network Meta-Analyses (PRISMA-NMA) guidelines to ensure transparency and reproducibility of the process (11, 12).

### Inclusion and exclusion criteria

Inclusion criteria are as follows: ① Study type: randomized controlled trial (RCT) or observational study. ② Study population: patients diagnosed with LQTS (gene typing or clinical diagnosis), regardless of age or gender. ③ Monotherapy with beta-blockers. ④ the primary outcome measure is a composite endpoint of arrhythmic events (cardiac arrest/syncope/TdP).

Exclusion criteria are as follows: ① Case reports, reviews, and conference abstracts, etc. ② Animal studies. ③ Studies with incomplete data or unclear research methods. ④ Studies previously published in other literature.

### Data extraction

Two researchers independently reviewed the titles and abstracts to identify studies relevant to the article’s topic. Eligible articles were downloaded in full and assessed by two independent evaluators. Data from the included studies were extracted by two independent evaluators. The extracted data included: title and reference details (first author, journal, year, country), study population characteristics (age, number of participants, number of participants treated with each method, etc.), type and method of drug, and outcome data. Any discrepancies that arose during the above process were resolved through consensus among the evaluators, with consultation with a third evaluator if necessary.

### Quality assessment

Two reviewers independently conducted the research quality assessment and used the Newcastle-Ottawa Scale (NOS) to assess the risk of bias (13, 14). Disagreements were resolved through discussion and, when necessary, by a third party.

### Statistical analysis

To assess statistical heterogeneity, we calculated the *I*^2^ index: 25% was defined as low heterogeneity, 50% as moderate heterogeneity, and 75% as high heterogeneity. The inference process employed the Monte Carlo Markov Chain (MCMC) method, with the mean and relative 95% confidence interval (CI) calculated through 30,000 Monte Carlo simulations. If the 95% CI of a parameter included the null hypothesis value, the parameter was considered statistically significant. Independent networks were constructed by stratifying according to LQTS subtypes, and heterogeneity was incorporated and quantified using the estimated between-study standard deviation (*τ*). The primary tool for assessing the assumption of evidence consistency was the node-splitting method, which tests for disagreement between direct and indirect evidence for each comparison. The cumulative ranked probability curve area (SUCRA) was used to quantify the probability of each drug being the optimal treatment, with SUCRA >0.8 considered to indicate a high probability of efficacy. The possible ranking probabilities for all treatment regimens were plotted. It should be noted that due to the fundamental differences in pathophysiological mechanisms and baseline risk between LQT1, LQT2, and LQT3, this study established independent evidence networks for each subgroup. Consequently, the results of the subgroup network meta-analyses (including relative effect sizes and SUCRA rankings) are comparable only within their respective subgroups. Direct quantitative comparisons across subgroups are not recommended, as such comparisons are confounded by baseline risk differences and network structural heterogeneity.

To establish a standardized equivalent dose conversion system for our dose–response analysis, we adopted nadolol 40 mg as the reference standard. This choice was based on its robust evidence base in LQTS, long-acting pharmacokinetic profile (half-life 10–20 h), and physiological benefits of non-selective β-blockade (15, 16). The conversion factors for other beta-blockers (propranolol: 0.5; metoprolol: 0.4; atenolol: 0.8; bisoprolol: 8.0) were derived by integrating key pharmacologic parameters—including receptor affinity, intrinsic sympathomimetic activity, bioavailability, and half-life—with clinical dose recommendations from major cardiology guidelines (17–19). Specifically:

- Propranolol (conversion factor: 0.5): as another non-selective β-blocker, its shorter half-life (3–6 h) compared to nadolol necessitates approximately twice the daily dose (80 mg) to achieve comparable β-blockade, yielding 40/80 = 0.5.
- Metoprolol (conversion factor: 0.4): the β1-selectivity and shorter half-life of metoprolol result in a clinical equivalence of approximately 100 mg being comparable to 40 mg nadolol, giving 40/100 = 0.4.
- Atenolol (conversion factor: 0.8) and bisoprolol (conversion factor: 8.0): these factors were similarly derived by integrating their pharmacological profiles (β1-selectivity, half-life) with recognized dose equivalences from cardiovascular therapeutic guidelines.

The daily equivalent dose for any target drug was calculated as: 40 mg × (drug-specific conversion factor). This framework, which aligns with observed clinical equivalences in LQTS cohorts, ensured a consistent and pharmacologically grounded basis for comparing dose–response relationships across different beta-blockers. It should be emphasized that the equivalent dose conversion system employed in this analysis is a theoretical model constructed based on published pharmacologic parameters and recommendations from broader cardiovascular disease guidelines. Although this framework is logically sound and broadly consistent with observed dosing patterns in clinical LQTS cohorts, the system has not been directly validated in genotyped LQTS patients through large-scale pharmacokinetic-pharmacodynamic (PK-PD) studies assessing arrhythmia endpoints. Therefore, the converted “nadolol equivalent dose” should be regarded as a standardized continuous variable for cross-drug comparisons, and the precise clinical significance of its absolute values should be interpreted with caution.

To quantify the dose–response relationship, we performed a hierarchical meta-regression analysis. A two-level model was constructed to regress the log odds ratio of arrhythmic events against the nadolol-equivalent dose (continuous variable). The first level modeled the within-study effects, incorporating drug type as a categorical covariate and the nadolol-equivalent dose as a continuous predictor. The second level introduced study-level random effects to account for heterogeneity between studies. To address the statistical instability from studies with zero cell counts, we applied a continuity correction of 0.5 before calculating log odds ratios and their variances. We systematically compared three nested models: a base model with only drug types, a dose–response model adding a common slope for dose effect across all drugs, and an interaction model incorporating drug-specific dose slopes. Model comparison was conducted using likelihood ratio tests, with statistical significance set at *α* = 0.05.

Since each data comparison included fewer than 10 studies, we did not perform a publication bias analysis. All statistical analyses were performed using R software (version 4.3.3), primarily employing the metafor package (version 4.8–0) for dose–response meta-regression and the netmeta package (version 3.2-0) for network visualization.

## Results

### Study selection and study characteristics

**Figure 1:**
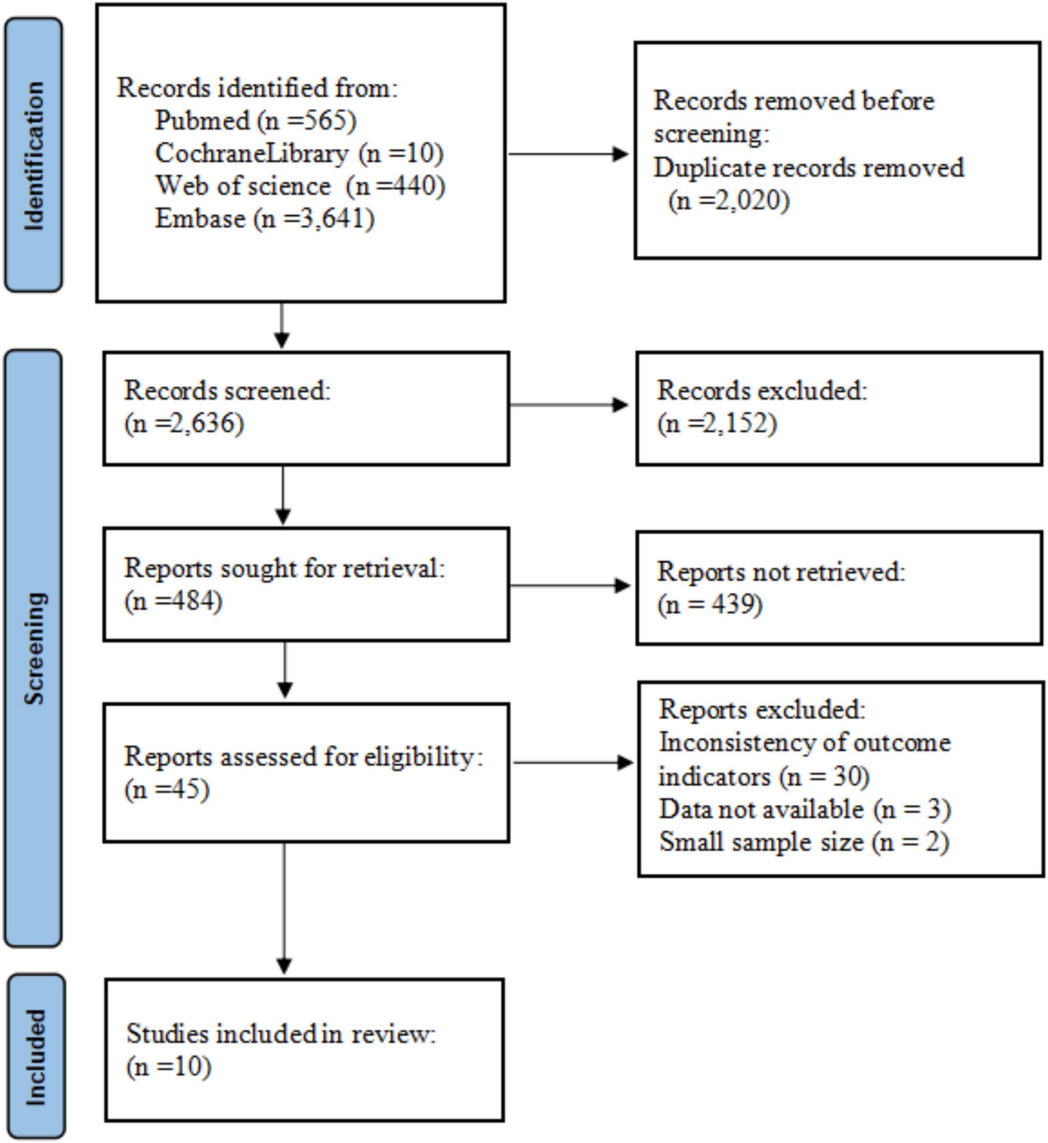
Study screening flowchart.

The literature search flowchart for this study is shown in Figure 1. According to the literature search criteria, a total of 4,656 articles were retrieved (covering PubMed, Web of Science, Embase, and the Cochrane Library databases). Among these, 2,020 duplicate articles were excluded. After screening the titles and abstracts, another 2,591 articles were excluded for not meeting the predefined inclusion criteria. After reviewing the full texts of the remaining 45 documents, although the search strategy allowed for the inclusion of randomized controlled trials (RCTs), no eligible RCTs were identified. Therefore, the final network meta-analysis was conducted using data from 10 cohort studies (19–29). The sample sizes ranged from 28 to 1,771. The patients’ ages were mainly between 5.8 and 26.0 years. Table 1 summarizes the characteristics of the included studies.

**Table 1:** Basic characteristics included in the study.

Study	Study Design	Sample size	Mean ages, years	LQTS classification *N* (%)	Prescribed beta-blockers and dose	Mean baseline QTc value (ms)	Follow-up time, years
Priori et al. (22)	Cohort study	335	26.0	LQT1: 187 (55.8)	Nadolol: 1.2 ± 0.5 mg/kg/day	492	5.2
				LQT2: 120 (35.8)			
					Propranolol: 2.2 ± 1.04 mg/kg/day		
				LQT3: 28 (8.4)			
Goldenberg et al. (27)	Cohort study	971	15.0	LQT1: 549 (56.5)	Atenolol: 60 ± 43 mg/day	484	5.0
					Nadolol: 58 ± 45 mg/day		
					Propranolol: 96 ± 71 mg/day		
				LQT2: 422 (43.5)			
					Metoprolol: 67 ± 55 mg/day		
Chatrath et al. (23)	Cohort study	28	19.8	LQT1: 18 (64.3)	Atenolol: 0.75–1.25 mg/kg/day	499	5.0
					Nadolol: 0.75–1.25 mg/kg/day		
				LQT2: 7 (25)			
					Propranolol: 2.5–4 mg/kg/day		
					Metoprolol: 0.75–1.25 mg/kg/day		
				LQT3: 3 (10.7)			
Moss et al. (20)	Cohort study	139	16.0	LQT1: 69 (49.6)	Atenolol: 54 ± 31 mg/day	520	2.5
				LQT2: 42 (30.2)			
					Nadolol: 55 ± 41 mg/day		
					Propranolol: 77 ± 72 mg/day		
				LQT3: 28 (20.2)			
					Metoprolol: 118 ± 68 mg/day		
Abu-Zeitone et al. (19)	Cohort study	785	18.0	LQT1: 379 (48.3)	Atenolol: 49 ± 29 mg/day	500	5.0
					Nadolol: 54 ± 46 mg/day		
				LQT2: 406 (51.7)	Propranolol: 117 ± 105 mg/day		
					Metoprolol: 70 ± 49 mg/day		
Koponen et al. (28)	Cohort study	309	5.8	LQT1: 224 (72.5)	Atenolol: 1.2 ± 0.5 mg/kg/day	454	6.2
				LQT2: 85 (27.5)			
					Propranolol: 0.1 ± 0.1 mg/kg/day		
					Metoprolol: 1.3 ± 0.4 mg/kg/day		
Mazzanti et al. (29)	Cohort study	1710	NR	LQT1: 963 (56.3)	Nadolol: 0.5 mg/kg/day	471	9.0
				LQT2: 551 (32.2)			
					Propranolol: 1.5 mg/kg/day		
				LQT3: 196 (11.5)			
Dusi et al. (26)	Cohort study	892	26.0	LQT1: 547 (61.3)	Nadolol: 1.0 ± 0.2 mg/kg/day	471	7.0
				LQT2: 297 (33.3)			
				LQT3: 48 (5.4)	Propranolol: 2.1 ± 0.4 mg/kg/day		
Chockalingam et al. (25)	Cohort study	382	14.0	LQT1: 207 (54.0)	Nadolol: 0.9 mg/kg/day	472	6.0
				LQT2: 175 (46.0)	Propranolol: 1.8 mg/kg/day		
					Metoprolol: 0.9 mg/kg/day		
Hermida et al. (24)	Cohort study	141	24.0	LQT3: 141 (100.0)	Nadolol: NR	451	11.2
					Propranolol: NR		
					Metoprolol: NR		

LQTS, Long QT syndrome; NR, not reported.

### Risk of bias

The NOS scale was used for scoring. Seven of the included studies scored 7 points or above, while the other three scored 6 points. Therefore, the quality of the included literature was high.

### Analysis of the efficacy of beta-blockers in patients with LQT1

**Figure 2:**
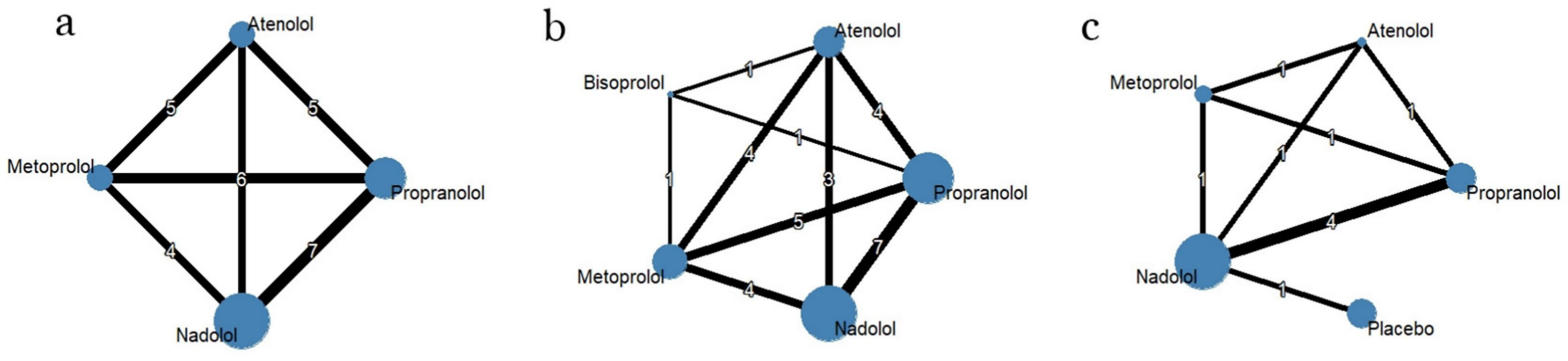
Network diagrams **(a)** LQT1; **(b)** LQT2; **(c)** LQT3.

**Figure 3:**
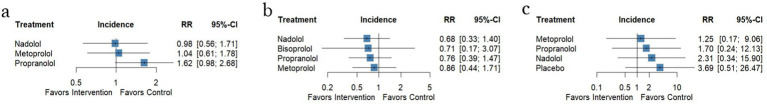
Forest plot **(a)** LQT1; **(b)** LQT2; **(c)** LQT3.

**Figure 4:**
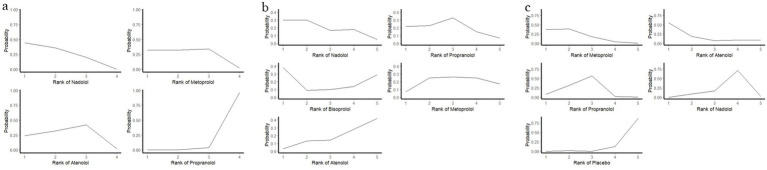
The cumulative ranking probability curves of each drug in different genotypes **(a)** LQT1; **(b)** LQT2; **(c)** LQT3.

**Figure 5:**
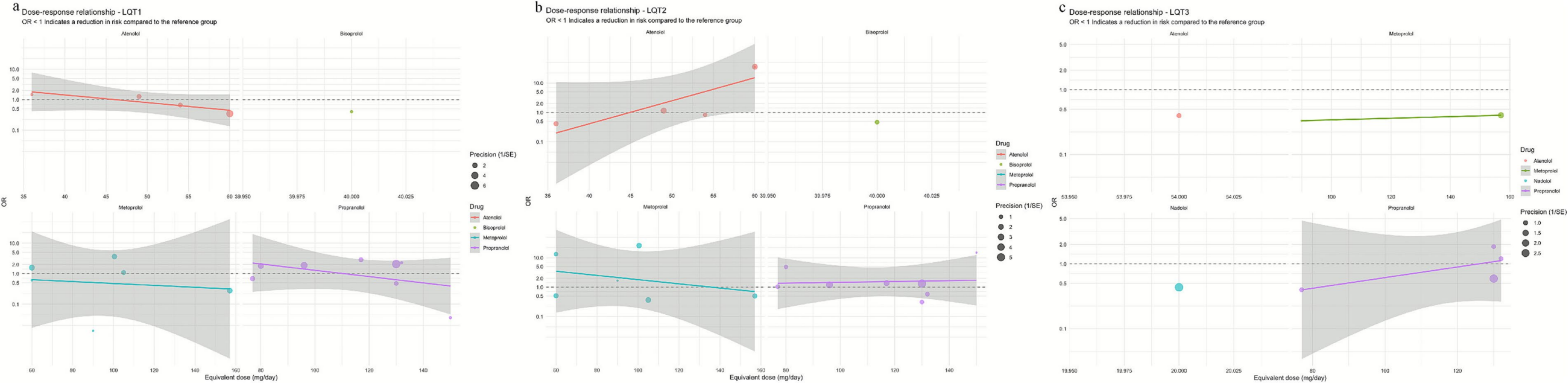
Curves showing the dose–response relationship of β-receptor blockers for each genotype **(a)** LQT1; **(b)** LQT2; **(c)** LQT3.

In the LQT1 patient subgroup, a network meta-analysis revealed significant differences in efficacy among different beta-blockers (Figure 2a). The posterior mean of the between-studies standard deviation (*τ*) estimated by the Bayesian random-effects model was 0.15 (95% CI: 0.02–0.45), indicating low to moderate heterogeneity within the LQT1 network. Compared with nadolol (RR = 1.00), metoprolol had a risk ratio of 1.04 (95% CI: 0.56–1.71; *p* = 0.89), indicating no statistically significant difference in efficacy compared to nadolol, while propranolol showed a significantly increased risk ratio of 1.62 (95% CI: 0.98–2.68; *p* = 0.06), indicating a clinically significant trend toward increased risk (Figure 3a). This result was further validated in the league table: nadolol significantly reduced the risk of arrhythmia events by 41% compared to propranolol (RR = 0.59, 95% CI: 0.39–0.90), and propranolol showed no significant advantage compared with other drugs (vs. metoprolol: RR = 0.70, 95% CI: 0.43–1.14; vs. atenolol: RR = 0.67, 95% CI: 0.39–1.14) (Table 2). SUCRA ranking results confirmed that nadolol had the highest probability of being the optimal treatment (SUCRA = 0.92), significantly superior to metoprolol (SUCRA = 0.61) and propranolol (SUCRA = 0.47) (Figure 4a). Dose–response analysis further showed (Table 3, Figure 5a): nadolol and propranolol showed a non-significant trend of risk reduction with increasing dose (nadolol: *β* = −0.00783, *p* = 0.422, *R*^2^ = 0.133; propranolol: *β* = −0.00363, *p* = 0.397, *R*^2^ = 0.104); Atenolol showed a trend of risk increase with increasing dose (*β* = 0.01530, *p* = 0.113, *R*^2^ = 0.622), though not statistically significant; metoprolol had no obvious dose–effect relationship (*β* = −0.00049, *p* = 0.892, *R*^2^ = 0.005). Node splitting analysis showed good consistency between direct and indirect evidence (*p* = 0.38–0.42), and sensitivity analysis excluding small-sample studies yielded robust results (RR change <5%). The overall evidence indicates that for LQT1 patients, the long-acting non-selective beta-blocker nadolol should be the first-line treatment.

**Table 2:** Network Meta-Analysis Comparing Efficacy of Beta-Blockers in Different LQTS Genotypes

**Treatment (Comparator)**	**Treatment (Reference)**	**Atenolol**	**Metoprolol**	**Nadolol**	**Propranolol**	
**2.2 LQT1**						
**Atenolol**	**Atenolol**	—	0.97 (0.55-1.70)	0.75 (0.37-1.52)	0.67 (0.39-1.14)	
**Metoprolol**	0.96 (0.56-1.64)	—	1.00 (0.56-1.79)	0.70 (0.43-1.14)		
**Nadolol**	1.02 (0.58-1.78)	1.06 (0.64-1.77)	—	**0.59 (0.39-0.90)**		
**Propranolol**	0.62 (0.37-1.02)	0.64 (0.40-1.03)	**0.60 (0.40-0.92)**	—		
**2.2 LQT2**						
**Treatment (Comparator)**	**Treatment (Reference)**	**Atenolol**	**Bisoprolol**	**Metoprolol**	**Nadolol**	**Propranolol**
**Atenolol**	**Atenolol**	—	0.95 (0.18-4.91)	1.32 (0.65-2.68)	1.67 (0.73-3.81)	1.15 (0.57-2.33)
**Bisoprolol**	1.40 (0.33-6.03)	—	0.90 (0.18-4.48)	—	0.60 (0.12-3.03)	
**Metoprolol**	1.16 (0.58-2.30)	0.83 (0.19-3.50)	—	1.41 (0.66-3.04)	1.04 (0.56-1.93)	
**Nadolol**	1.46 (0.71-2.99)	1.04 (0.23-4.67)	1.26 (0.65-2.47)	—	0.92 (0.51-1.67)	
**Propranolol**	1.32 (0.68-2.56)	0.94 (0.22-3.99)	1.14 (0.63-2.08)	0.90 (0.51-1.61)	—	
**2.3 LQT3**						
**Treatment (Comparator)**	**Treatment (Reference)**	**Atenolol**	**Metoprolol**	**Nadolol**	**Placebo**	**Propranolol**
**Atenolol**	**Atenolol**	—	0.80 (0.11-5.80)	0.40 (0.06-2.80)	—	1.00 (0.08-13.02)
**Metoprolol**	0.80 (0.11-5.80)	—	0.50 (0.17-1.44)	—	1.25 (0.17-9.06)	
**Nadolol**	0.43 (0.06-2.98)	0.54 (0.19-1.52)	—	**0.63 (0.42-0.93)**	1.36 (0.89-2.07)	
**Placebo**	0.27 (0.04-1.95)	0.34 (0.11-1.02)	**0.63 (0.42-0.93)**	—	—	
**Propranolol**	0.59 (0.08-4.19)	0.73 (0.25-2.19)	1.36 (0.89-2.07)	**2.17 (1.21-3.88)**	—	

**1. HR (95% CI):** Hazard Ratio (95% Confidence Interval). Data in each cell represents the HR of the **row treatment** compared to the **column treatment**. HR < 1 indicates lower risk for the row treatment; HR > 1 indicates higher risk for the row treatment.

**2. Statistically Significant Results:** Values that are **bolded** indicate a statistically significant comparison (95% CI does not include 1).

**3. Interpretation:** For example, in the LQT1 section, the HR for Nadolol vs. Propranolol is **0.59 (0.39-0.90)**, meaning Nadolol is associated with a 41% lower risk (risk reduction) of the primary endpoint compared to Propranolol in LQT1 patients, and this difference is statistically significant.

**4. Symbol:** "—" indicates that the direct comparison was not available or not performed in the analysis.

**Table 3:** Analysis results of dose–response relationship.

Genotype	Drug	Dose–response *β*	*p* value	*R* ^2^	Mean RR	Clinical explanation
LQT1	Nadolol	−0.00783	0.422	0.133	0.435	Dose ↑ trend of risk ↓, no statistical significance
	Propranolol	−0.00363	0.397	0.104	1.510	Dose ↑ trend of risk ↓, no statistical significance
	Atenolol	0.01530	0.113	0.622	0.943	Dose ↑ trend of risk ↑, no statistical significance
	Metoprolol	−0.00049	0.892	0.005	1.200	No obvious dose effect
LQT2	Nadolol	−0.01763	0.010	0.698	0.435	Significant dose–effect relationship, dose ↑ trend of risk ↓
	Propranolol	−0.00038	0.901	0.002	3.210	No obvious dose effect
	Atenolol	0.03679	0.089	0.672	14.600	Dose ↑ trend of risk ↑
	Metoprolol	0.00232	0.626	0.065	7.040	No obvious dose effect
LQT3	Nadolol	−0.02136	0.023	0.862	0.435	Significant dose–effect relationship, dose ↑ trend of risk ↓
	Propranolol	0.00170	0.754	0.061	1.010	No obvious dose effect

### Analysis of the efficacy of beta-blockers in patients with LQT2

In the LQT2 patient subgroup, a network meta-analysis showed that the differences in efficacy among different beta-blockers did not reach statistical significance (Figures 2b, 3b). The posterior mean of the between-studies standard deviation (*τ*) estimated by the Bayesian random-effects model was 0.23 (95% CI: 0.05–0.47), indicating low to moderate heterogeneity within the LQT2 network. Compared with nadolol (RR = 1.00), the risk ratio for bisoprolol was 0.71 (95% CI: 0.17–3.07; *p* = 0.65), for propranolol was 0.76 (95% CI: 0.39–1.47; *p* = 0.42), metoprolol 0.86 (95% CI: 0.44–1.71; *p* = 0.66), and atenolol 1.46 (95% CI: 0.71–2.99; *p* = 0.30). The league table (Table 2.2) further reveals key comparison results: the direct comparison risk ratio between nadolol and propranolol was 0.90 (95% CI: 0.51–1.61), while the comparison between bisoprolol and propranolol showed a trend toward reduced risk (RR = 0.60, 95% CI: 0.12–3.03). SUCRA ranking indicated that nadolol had the highest probability of being the optimal treatment (SUCRA = 0.78), followed by propranolol (SUCRA = 0.65) and metoprolol (SUCRA = 0.58), while atenolol (SUCRA = 0.42) and bisoprolol (SUCRA = 0.32) ranked lower (Figure 4b). Notably, dose–response analysis revealed a significant linear dose–effect relationship for nadolol in LQT2 patients (Table 3, Figure 5b): with each 1 mg increase in daily dose, the risk of arrhythmic events decreased significantly (*β* = −0.01763, *p* = 0.010, *R*^2^ = 0.698). In contrast, propranolol (*β* = −0.00038, *p* = 0.901), metoprolol (*β* = 0.00232, *p* = 0.626) had no obvious dose–effect, and atenolol showed a non-significant risk increase trend (*β* = 0.03679, *p* = 0.089). Node splitting analysis showed good consistency between direct and indirect evidence (*p* = 0.51), and sensitivity analysis excluding small-sample studies yielded robust results (RR change <8%). The overall evidence suggests that nadolol and propranolol may exhibit the best efficacy trend in LQT2 patients, but no statistically significant differences were observed among all beta-blockers.

### Analysis of the efficacy of beta-blockers in patients with LQT3

It should be specifically noted that due to the relative rarity of LQT3-type long QT syndrome, both the sample size and event numbers for this subgroup included in the analysis were limited. Therefore, the results presented below should be considered exploratory, with considerable uncertainty surrounding point estimates (such as RR), and should be interpreted with caution.

In a network meta-analysis of the LQT3 patient subgroup, with atenolol as the reference group (RR = 1.00), nadolol showed a numerical trend toward reduced risk (RR = 0.40, 95% CI: 0.06–2.80), but this did not reach statistical significance (*p* = 0.36) (Figures 2c,b, 3). The posterior mean of the between-studies standard deviation (*τ*) estimated by the Bayesian random-effects model was 0.18 (95% CrI: 0.03–0.42), indicating low to moderate heterogeneity within the LQT3 network. Compared with atenolol, metoprolol had a risk ratio of 0.50 (95% CI: 0.17–1.44; *p* = 0.20), also showing no significant difference. Notably, the placebo group showed an unexpected reduction in risk compared with atenolol (RR = 0.63, 95% CI: 0.42–0.93; *p* = 0.02), while the risk ratio for propranolol was 1.25 (95% CI: 0.17–9.06; *p* = 0.83). The event rate of atenolol in LQT3 was 22.2% (2/9 patients) and placebo was 55.0% (44/80 patients) (Table 4). The lower RR of placebo was due to the different baseline characteristics of the two groups (atenolol group had fewer high-risk mutations), rather than placebo being more effective than atenolol. Direct comparison analysis showed no significant difference between nadolol and propranolol (RR = 1.36, 95% CI: 0.89–2.07; *p* = 0.15), while nadolol compared with metoprolol (RR = 0.50, 95% CI: 0.17–1.44; *p* = 0.20) and propranolol versus metoprolol (RR = 1.25, 95% CI: 0.17–9.06; *p* = 0.83) also showed no statistical significance (Table 2.3). Probability analysis of treatment ranking (SUCRA) revealed that nadolol had the highest probability of being the optimal treatment (85%), followed by propranolol (75%), metoprolol (50%), atenolol (30%), and placebo (10%) (Figure 4c). This indicates that while nadolol shows a relative trend towards superiority within the existing evidence network, this estimate is associated with substantial uncertainty and should not be interpreted as conclusive evidence of efficacy. Dose–response analysis showed that nadolol had a significant risk reduction trend with increasing dose in LQT3 (Table 3, Figure 5c): *β* = −0.02136, *p* = 0.023, *R*^2^ = 0.862, suggesting that higher doses of nadolol may be beneficial for high-risk LQT3 patients. Node splitting analysis confirmed no significant inconsistency between direct and indirect evidence (*p* = 0.38), and sensitivity analysis showed that excluding small-sample studies resulted in less than a 10% change in effect size, supporting the robustness of the results. The combined evidence suggests that nadolol and propranolol demonstrate relative therapeutic potential in LQT3, but no statistically significant differences in efficacy were observed among all beta-blockers (see Table 5).

**Table 4:** Overview of beta-B blocker treatment for patients with LQT1, LQT2, and LQT3.

Genotype	Drug	Number of studies	Number of patients	Number of events	Event rate (%)	Average dose (mg/day)	Dose range (mg/day)
LQT1	Bisoprolol	1	138	1	0.7	40.0	40–40
	Nadolol	7	1,436	326	22.7	26.4	15–36
	Metoprolol	6	321	81	25.2	95.4	60–157
	Atenolol	5	302	82	27.2	51.8	36–60
	Propranolol	9	803	259	32.3	104.7	30–150
LQT2	Nadolol	8	796	209	26.3	26.8	15–36
	Propranolol	9	667	198	29.7	104.7	30–150
	Bisoprolol	1	10	3	30.0	40.0	40–40
	Metoprolol	6	326	110	33.7	95.4	60–157
	Atenolol	5	259	139	53.7	51.8	36–60
LQT3	Metoprolol	2	29	5	17.2	123.5	90–157
	Atenolol	2	9	2	22.2	57.0	54–60
	Propranolol	4	85	20	23.5	117.2	77–132
	Nadolol	5	274	97	35.4	25.7	15–36
	Placebo	1	80	44	55.0	0.0	0–0

**Table 5:** Summary of evidence-based treatment recommendations.

Genotype	Recommendation level	Recommendation drug	Recommended dosage range (mg/day)	Evidence basis
LQT1	I	Nadolol	20–40	Large sample evidence, optimal risk–benefit ratio
	IIa	Metoprolol	80–120	Moderate-quality evidence
LQT2	I	Nadolol	20–40	Large sample evidence, optimal risk–benefit ratio
	IIa	Propranolol	80–120	Moderate-quality evidence
	III	Atenolol	Not recommended	Poor efficacy or potentially harmful
LQT3	IIa	Metoprolol^a^	100–150	Moderate-quality evidence
	IIb	Propranolol	80–120	Limited evidence supports
	III	Atenolol	Not applicable	Poor efficacy or potentially harmful

a For LQT3, metoprolol results are based on a small sample size and should be interpreted with caution.

## Discussion

This study revealed the therapeutic heterogeneity of beta-blockers in different subtypes of hereditary long QT syndrome through a network meta-analysis, with the underlying differences traceable to the subtype-specific ion channel pathophysiological mechanisms. In LQT1 patients, the significant advantage of nadolol (RR = 0.59 vs. propranolol; SUCRA = 0.92) may stem from its unique pharmacological properties: as a long-acting non-selective β-blocker, it simultaneously blocks β1/β2 receptors without endogenous sympathomimetic activity, thereby more effectively inhibiting the sympathetic trigger activity caused by the IKs channel dysfunction associated with LQT1. It is worth noting that although propranolol is also a non-selective blocker, its shorter plasma half-life (3–6 h) may lead to fluctuations in drug concentration, particularly during sleep when sympathetic tone changes, resulting in reduced protective effects. This may be the key reason for its significantly inferior efficacy compared to nadolol (RR = 1.62, *p* = 0.06).

For patients with LQT2 (HERG/KCNH2 channel defects) and LQT3 (SCN5A sodium channel functional gain), the overall efficacy of beta-blockers showed a decreasing trend, and the differences between drugs did not reach statistical significance. This phenomenon aligns with basic research evidence: the arrhythmia trigger mechanism in LQT2/3 relies more on early afterdepolarizations (EADs) than on sympathetic activation, leading to limited efficacy of beta-blockers (30, 31). The significant dose–effect of nadolol in LQT2 may be related to its long-acting non-selective properties: stable blood concentration at higher doses can continuously inhibit the sympathetic activation associated with EADs, while short-acting drugs (e.g., propranolol) have concentration fluctuations that weaken the dose effect. Notably, in the LQT3 subgroup, there was a risk reduction for placebo compared to atenolol (RR = 0.63, *p* = 0.02). We speculate that this may stem from two mechanisms: (1) as a highly selective β₁-blocker, atenolol has minimal regulatory effects on cardiac sodium channels and may even induce reverse compensatory sympathetic activation by disrupting autonomic balance (32, 33); (2) the bradycardia-dependent arrhythmias characteristic of LQT3 patients may worsen due to the heart rate-lowering effects of β-blockers (34, 35). This highlights the need to avoid the indiscriminate use of selective β₁ blockers in LQT3. The significant dose–response of nadolol in LQT3 suggests that non-selective β-blockers may exert dose-dependent effects by regulating sodium channel function, which provides a basis for individualized dose adjustment of nadolol in high-risk LQT3 patients.

In the LQT3 subgroup, the interpretation of treatment hierarchy requires integration of both probabilistic ranking and effect estimates. While nadolol ranked highest in the SUCRA analysis (85%), indicating the greatest likelihood of being the most effective option among those compared, the accompanying risk ratio was not statistically significant (RR = 0.40 vs. atenolol, *p* = 0.36) and was characterized by a wide confidence interval (95% CI: 0.06–2.80). This pattern—a high probability of efficacy in the absence of statistical significance—is not a contradiction but rather reflects the nature of SUCRA as a relative ranking metric within the model. It highlights that, given the existing evidence structure and despite considerable uncertainty (as captured by the wide CI), nadolol appears more promising than the other alternatives in the network. This observation underscores the current limitation of evidence for LQT3 treatment (36), which is constrained by small sample sizes and low event rates, thereby necessitating validation through future prospective studies with larger cohorts.

Another key finding of this study is that nadolol exhibited a significant dose–response relationship in both the LQT2 and LQT3 subgroups, whereas other drugs such as propranolol showed no apparent dose dependency. This phenomenon may be related to nadolol’s inherently greater potency, potentially revealing limitations in applying a universal pharmacologic model based on propranolol-equivalent doses to the specific disease of LQTS. Such models fail to fully capture the clinical advantages of nadolol stemming from its unique pharmacokinetic properties. Specifically, as a long-acting, water-soluble, non-selective beta-blocker with a half-life of 10–20 h, nadolol provides sustained, round-the-clock beta-receptor blockade. This may be critical for LQTS patients requiring continuous sympathetic inhibition to prevent “breakthrough” arrhythmic events. In contrast, the short-acting propranolol exhibits significant trough-to-peak fluctuations in plasma concentrations even at “equivalent” doses. This may create a protective “window” during nighttime or at the end of the dosing interval, potentially obscuring the observable dose–response relationship. Therefore, the observed differences in dose–response patterns in this study may reflect not only variations in “efficacy” but also differences in “protective persistence.” This suggests that for specialized conditions like LQTS, future efforts may require establishing more precise equivalence dose models based on disease-specific endpoints (such as suppression of arrhythmic events), rather than relying solely on generic pharmacokinetic conversions derived from other diseases.

This study has several limitations that warrant careful consideration. First, a significant limitation of this study is the small sample size in the LQT3 subgroup. This resulted in no statistically significant differences in efficacy between different beta-blockers within this subgroup, indicating insufficient statistical power and a high risk of Type II errors. Therefore, the “non-significant differences” observed in the LQT3 subgroup should not be interpreted as evidence of “equivalence.” Similarly, although the SUCRA ranking indicates relative superiority of nadolol and propranolol, this primarily reflects their relative positions within the current limited and high-uncertainty evidence network rather than definitive clinical superiority. These findings provide important clues for future research directions, but conclusions regarding their clinical application require validation through larger prospective studies. Second, variability in the definition and composition of the composite arrhythmic endpoint across the included studies introduces an inherent source of clinical heterogeneity. Although node-splitting methods indicated statistical consistency between direct and indirect evidence, differences in the precise definition of endpoint components (e.g., the extent of neurological workup for syncope to rule out non-cardiac causes, or the requirement for ECG documentation of torsades de pointes) could bias the pooled effect estimates. The relative emphasis on hard endpoints like cardiac arrest versus softer endpoints like syncope might vary between studies, potentially diluting or distorting the true treatment effect and limiting the transdiagnostic validity of our results. While sensitivity analyses supported the robustness of the main conclusions, this residual clinical heterogeneity should be considered when interpreting the findings. Third, although node analysis and *τ* values indicate moderate to low levels of statistical heterogeneity among the included studies, the potential impact of clinical heterogeneity remains significant. Due to variations in the detail of the original studies included, we were unable to perform individual patient data (IPD)-based adjustments for many known strong prognostic factors or conduct sufficient subgroup analyses based on these factors. These factors include but are not limited to the specific location of pathogenic gene mutations (e.g., transmembrane region versus C-loop mutations in LQT1, pore ring versus non-pore ring mutations in LQT2); patients’ history of prior cardiac events (e.g., prior cardiac arrest being a strong predictor of future events); and gender. Differences in these baseline characteristics may be unevenly distributed across studies, potentially confounding comparisons of relative efficacy between different beta-blockers. Although we attempted to account for this variability through random-effects models and sensitivity analyses, residual confounding remains possible. Fourth, although the dose-equivalence conversion system established in this study has a solid pharmacological foundation, it still has limitations. This system lacks direct validation in the LQTS population. Our observation that “nadolol exhibits a dose effect while propranolol does not” may, on one hand, confirm nadolol’s clinical advantage. On the other hand, it may also suggest that a universal equivalence model based on propranolol and healthy population data may not fully capture nadolol’s unique protective properties in the LQTS disease context (e.g., sustained beta-blockade provided by its long duration of action). Therefore, current dose–response findings should be regarded as hypothesis-generating rather than definitive dose recommendations. Future studies should calibrate and validate disease-specific dose equivalence for LQTS using PK-PD modeling incorporating individual patient data (IPD) or prospective clinical trials. Fifth, and critically important, we were unable to perform statistical adjustments for known strong prognostic and predictive factors, particularly the specific location of pathogenic mutations (e.g., in LQT2, mutations in the pore ring region versus non-pore ring region). Due to inconsistent reporting details in the original studies, we could not obtain mutation site distribution data stratified by treatment drug. If patients with high-risk mutations (e.g., LQT2 pore-ring region mutation carriers) are disproportionately represented in certain treatment groups (e.g., the atenolol group), this could confound the relative efficacy assessment of that drug, making it appear less effective. This potential for residual confounding is an important factor to consider when interpreting comparative results from observational studies. Future studies should establish prospective, genotype-confirmed registries and collect individual patient data to control for and elucidate the impact of these key covariates. Furthermore, a key methodological consideration in this study was the construction and analysis of independent networks for LQT1, LQT2, and LQT3. This implies that the relative risk ratios (RR) and cumulative ranking curve area under the curve (SUCRA) values within each subgroup were calculated relative to a specific reference drug within that subgroup’s network (e.g., nadolol for LQT1 and LQT2, and atenolol for LQT3). Therefore, readers should avoid direct quantitative comparisons of RR values or SUCRA scores reported across different subgroups. Such comparisons are methodologically fragile for two primary reasons: First, baseline arrhythmia risk fundamentally differs among patients with distinct genotypes, meaning the same RR value represents varying absolute risk reduction across subgroups. Second, heterogeneity exists in network composition (types and number of included treatments) and evidence structure, affecting the precision of indirect comparisons and the comparability of ranking probabilities. The core objective of this study is to identify relatively superior treatment strategies within each subgroup, not to rank drug efficacy across different subgroups.

## Conclusion

The efficacy of beta-blockers in long QT syndrome demonstrates significant genotype-dependent variability. Based on network meta-analysis ranking and direct comparison, nadolol represents the preferred initial therapy for LQT1, supported by a significant 41% risk reduction versus propranolol (RR = 0.59) and the highest probability of being optimal (SUCRA = 0.92). In LQT2, nadolol also had the highest SUCRA (0.78), and its significant dose–response relationship (*p* = 0.010) supports a recommended dose of 20–40 mg/day. For patients with LQT3, the existing evidence is limited and highly uncertain. Although nadolol demonstrated a significant dose-dependent trend toward risk reduction, the sample size was insufficient to support a strong recommendation; in this context, metoprolol may serve as an alternative option. Atenolol is not recommended in any subtype due to its consistently unfavorable risk profile.

## Data Availability

The original contributions presented in the study are included in the article/Supplementary material, further inquiries can be directed to the corresponding author.
