# Supplementary material for: Different beta-blockers for preventing arrhythmic events in patients with long QT syndrome: a network meta-analysis
Source: Front Med (Lausanne). 2025 Dec 16;12:1730755. doi: 10.3389/fmed.2025.1730755 (PMC12748156; doi:10.3389/fmed.2025.1730755)
Supplement: Supplementary file 1 [file Table_1.docx]

**Supplementary Table**

**Table S1**. Search strategy used for all searched databases

Literature search in PubMed, Web of Science, Embase and The Cochrane Library.

| **Databases** | **Group** | **Search Syntax** | **Records** |
| --- | --- | --- | --- |
| **PubMed** | #1 | "Long QT Syndrome"[Mesh] OR "Electrocardiogram QT Prolonged"[Title/Abstract] OR "LQTS"[Title/Abstract] | 10299 |
|  | #2 | "Adrenergic beta-Antagonists"[Mesh] OR "Adrenergic beta-Receptor Blockader"[Title/Abstract] OR "beta-Adrenergic Blocking Agents"[Title/Abstract] OR "beta-Adrenergic Blockers"[Title/Abstract] OR "beta blocker"[Title/Abstract] | 52958 |
|  | #3 | "Arrhythmias, Cardiac"[Mesh] OR "Arrhythmia"[Title/Abstract] OR "Cardiac Arrhythmia"[Title/Abstract] | 276387 |
|  | #4 | #1 AND #2 AND #3 | 565 |
| **Web of Science** | #1 | TS=("Long QT Syndrome" OR "Electrocardiogram QT Prolonged" OR "LQTS" ) | 18304 |
|  | #2 | TS=("Adrenergic beta-Antagonists" OR "Adrenergic beta-Receptor Blockader" OR "beta-Adrenergic Blocking Agents" OR "beta-Adrenergic Blockers" OR "beta blocker") | 65339 |
|  | #3 | TS=("Arrhythmias, Cardiac" OR "Arrhythmia" OR "Cardiac Arrhythmia") | 190106 |
|  | #4 | #1 AND #2 AND #3 | 440 |
| **Embase** | #1 | 'long qt syndrome'/exp | 16051 |
|  | #2 | 'long q-t syndrome':ti,ab,kw OR 'lqts':ti,ab,kw OR 'long qt syndrome':ti,ab,kw | 10440 |
|  | #3 | #1 OR #2 | 17786 |
|  | #4 | 'beta adrenergic receptor blocking agent'/exp | 378628 |
|  | #5 | 'adrenergic beta antagonists':ti,ab,kw OR 'adrenergic beta-antagonists':ti,ab,kw OR 'antiadrenergics, beta blocking':ti,ab,kw OR 'beta adrenergic antagonist':ti,ab,kw OR 'beta adrenergic blocker':ti,ab,kw OR 'beta adrenergic blockers':ti,ab,kw OR 'beta adrenergic blocking agent':ti,ab,kw OR 'beta adrenergic blocking drug':ti,ab,kw OR 'beta adrenergic receptor antagonist':ti,ab,kw OR 'beta adrenergic receptor blocker':ti,ab,kw OR 'beta adrenoceptor antagonist':ti,ab,kw OR 'beta adrenoceptor blocker':ti,ab,kw OR 'beta adrenoceptor blocking agent':ti,ab,kw OR 'beta adrenoceptor blocking drug':ti,ab,kw OR 'beta adrenolytic':ti,ab,kw OR 'beta adrenolytic agent':ti,ab,kw OR 'beta antagonist':ti,ab,kw OR 'beta antiadrenergic agent':ti,ab,kw OR 'beta blocker':ti,ab,kw OR 'beta blocking adrenergic agent':ti,ab,kw OR 'beta blocking agent':ti,ab,kw OR 'beta blocking drug':ti,ab,kw OR 'beta receptor adrenergic blocking agent':ti,ab,kw OR 'beta receptor blocker':ti,ab,kw OR 'beta receptor blocking agent':ti,ab,kw OR 'beta sympathicolytic agent':ti,ab,kw OR 'beta sympathicolytics':ti,ab,kw OR 'beta sympatholytic agent':ti,ab,kw OR 'betasympatholytic agent':ti,ab,kw OR 'beta adrenergic receptor blocking agent':ti,ab,kw | 22881 |
|  | #6 | #4 OR #5 | 380207 |
|  | #7 | 'heart arrhythmia'/exp | 790392 |
|  | #8 | 'arrhythmia':ti,ab,kw OR 'arrhythmias, cardiac':ti,ab,kw OR 'arrhytmia, heart':ti,ab,kw OR 'cardiac arrhythmia':ti,ab,kw OR 'cardiac arrhythmias':ti,ab,kw OR 'cardiac arrythmia':ti,ab,kw OR 'cardiac disrhythmia':ti,ab,kw OR 'cardiac dysrhythmia':ti,ab,kw OR 'cardial arrhythmia':ti,ab,kw OR 'ectopic heart rhythm':ti,ab,kw OR 'ectopic rhythm':ti,ab,kw OR 'heart aberrant conduction':ti,ab,kw OR 'heart arrhytmia':ti,ab,kw OR 'heart arrythmia':ti,ab,kw OR 'heart dysrhythmia':ti,ab,kw OR 'heart ectopic beat':ti,ab,kw OR 'heart ectopic ventricle contraction':ti,ab,kw OR 'heart rhythm disease':ti,ab,kw OR 'heart rhythm disorder':ti,ab,kw OR 'heart rhythm problem':ti,ab,kw OR 'myocardial arrhythmia':ti,ab,kw OR 'heart arrhythmia':ti,ab,kw | 121011 |
|  | #9 | #7 OR #8 | 806508 |
|  | #10 | #3 AND #6 AND #9 | 3641 |
| **Cochrane Library** | #1 | MeSH descriptor: [Long QT Syndrome] explode all trees | 442 |
|  | #2 | ("Electrocardiogram QT Prolonged" OR "LQTS"):ti,ab,kw | 46 |
|  | #3 | #1 OR #2 | 467 |
|  | #4 | MeSH descriptor: [Adrenergic beta-Antagonists] explode all trees | 5670 |
|  | #5 | ("Adrenergic beta-Receptor Blockader" OR "beta-Adrenergic Blocking Agents" OR "beta-Adrenergic Blockers" OR "beta blocker"):ti,ab,kw | 4034 |
|  | #6 | #4 OR #5 | 8284 |
|  | #7 | MeSH descriptor: [Arrhythmias, Cardiac] explode all trees | 13947 |
|  | #8 | ("Arrhythmia" OR "Cardiac Arrhythmia"):ti,ab,kw | 10906 |
|  | #9 | #7 OR #8 | 20926 |
|  | #10 | #3 AND #6 AND #9 | 10 |
